# Proceedings of the Fox-River Medical Society

**Published:** 1850-03

**Authors:** A. Danforth

**Affiliations:** Sec’y.


### ARTICLE IX.

*Proceedings of the Fox-River Medical Association*—At a Special Meeting held at Elgin, Kane Co., Ill., Feb. 1st, 1850.

Association met pursuant to adjournment. President Howland being absent, Vice-President Hale, of Dundee, was called to the Chair. The Roll being called, Drs. W. Danforth, O. Harvey, L. Hale, A. Danforth. N. Hard, and L. F. Torrey, answered to the call.

The minutes of the previous meeting, were then read and adopted. Addresses being called for, and Prof. S. G. Armor (who was expected to address the Association,) being absent, an invitation was extended to Prof. N. Hard, M.D., who accepted the same, and delivered an able and interesting address upon "Cholera," showing its contagious character as exhibited in the Epidemic of 1849, especially in that which appeared at Aurora, Kane, Co., Ill., the fallacy of "Specific Cures," and the departures from the usual "concomitant symptoms," as there exhibited.

*On Motion*, The order of business was suspended, and opportunity given for those present, (not members,) to become so if desired; whereupon, numerous Physicians, availed themselves of the opportunity, and became members, by complying with the requisitions of the Association.

The following Resolutions were then read and unanimously adopted.

*Resolved:* That the subject for discussion at the next meeting of the Association, shall be the Contagious Nature of Cholera.

*Resolved:* That this Association recommend to its members, and the Profession generally, frequent *Post-Mortem* Examinations, as a means of dispelling public prejudice, and affording valuable contributions to "Pathological Auatomy."

*Resolved:* That the Proceedings of this Meeting be published in the North Western Med. and Surg. Journal, and the Chicago and Fox-River papers.

*On Motion:* The Association adjourned to meet at the "Howard House," in St. Charles, Kane Co., Ill., on the fifth of March next. A numerous attendance on the part of the Profession is solicited.

A. DANFORTH, M.D., Sec'y.

